# Comparative transcriptome analysis by RNAseq of necrotic enteritis *Clostridium perfringens* during *in vivo* colonization and *in vitro* conditions

**DOI:** 10.1186/s12866-016-0792-6

**Published:** 2016-08-12

**Authors:** Valeria R. Parreira, Kay Russell, Spiridoula Athanasiadou, John F. Prescott

**Affiliations:** 1 Department of Pathobiology, University of Guelph, Guelph, ON N1G 2W1 Canada; 2 Disease Systems, Animal and Veterinary Sciences SRUC, Roslin Institute Building, Midlothian, EH25 9RG Scotland

**Keywords:** *Clostridium perfringens*, Transcriptome, RNASeq

## Abstract

**Background:**

Necrotic enteritis (NE) caused by *netB*-positive type A *Clostridium perfringens* is an important bacterial disease of poultry. Through its complex regulatory system, *C. perfringens* orchestrates the expression of a collection of toxins and extracellular enzymes that are crucial for the development of the disease; environmental conditions play an important role in their regulation. In this study, and for the first time, global transcriptomic analysis was performed on ligated intestinal loops in chickens colonized with a *netB*-positive *C. perfringens* strain, as well as the same strain propagated in vitro under various nutritional and environmental conditions.

**Results:**

Analysis of the respective pathogen transcriptomes revealed up to 673 genes that were significantly expressed in vivo. Gene expression profiles in vivo were most similar to those of *C. perfringens* grown in nutritionally-deprived conditions.

**Conclusions:**

Taken together, our results suggest a bacterial transcriptome responses to the early stages of adaptation, and colonization of, the chicken intestine. Our work also reveals how *netB*-positive *C. perfringens* reacts to different environmental conditions including those in the chicken intestine.

**Electronic supplementary material:**

The online version of this article (doi:10.1186/s12866-016-0792-6) contains supplementary material, which is available to authorized users.

## Background

*Clostridium perfringens* is an important pathogen of humans and animals. *netB*-positive type A strains of *C. perfringens* cause necrotic enteritis (NE) in broiler chickens, a common bacterial infection that has conventionally been controlled by antibiotics. However the removal of “growth-promoting” antibiotics in broiler chickens in Europe and increasing demands elsewhere for antibiotic-free chicken are focusing efforts to find alternative approaches to control [1, 2]. For this reason, in recent years there has been considerable effort to understand the pathogenesis of NE in the chicken, and numerous advances have been made [3–5]. Providing effective alternatives to antibiotics for control of NE may be facilitated through detailed understanding of gene expression by *C. perfringens* during the pathogenesis of NE.

Gene expression is a highly dynamic process controlled by a regulatory system that selectively turns genes on and off depending on a wide range of factors, including growth stage, environmental conditions, and stress situations. Regulation of gene expression allows organisms to adapt to their environment including their host [6]. High-throughput DNA sequencing methods have provided a comprehensive method for mapping and quantifying the complete set of transcripts (“transcriptome”) of an organism. This RNA sequencing method (“RNASeq”) has clear advantages over previous approaches to gene expression analysis [7].

The purpose of the study described here was to analyze differential expression of a necrotic enteritis *C. perfringens* strain under different environmental conditions including ligated intestinal loops in the chicken. Overall, this approach revealed global mechanisms employed by *netB*-positive *C. perfringens* to adapt to different environments and provides insight into virulence gene expression.

## Methods

### Bacterial strain and growth media

A *netB*-positive *C. perfringens* strain, CP1, with confirmed ability to reproduce NE experimentally [8] and to be transformed, was grown overnight at 37 °C under anaerobic conditions (80 % N_2_, 10 % H_2_, 10 % CO_2_) in TPG broth (5 % tryptone, 0.5 % protease peptone, 0.4 % glucose, 0.1 % thioglycollic acid; Difco Laboratories, Detroit, MI).

### In vitro experiments

To analyze the impact of environmental changes in *C. perfringens* gene expression and to compare these to conditions in vivo [IV], in vitro experiments were designed to induce nutritional changes and osmotic shock. For this purpose, 2 ml cultures were incubated at 37 °C until OD_600_ was 0.4–0.5, and then collected by centrifugation at 4000 x g for 2 min. Pelleted cells were then transferred to three different conditions: (i) nutrient-poor media condition [PM], by addition of an equal volume of peptone-protease water [9], (ii) to osmotic shock condition [OS], by adding to an equal volume of TPG supplemented with 1.5 % NaCl, and (iii) for the control group, cells were transferred to fresh TPG (rich media condition) [RM]. After incubating for 1 h at 37 °C under anaerobic conditions RNAlater (LifeTechnologies, Burlington, ON) was added to stabilize total RNA and stored at 4 °C overnight. All experiments were performed in three biological replicates.

### Chicken intestinal loop assay

All animal experiments were performed with the approval of the Institutional Animal Care and Use Committee (AU AE 40–2013) given to Dr. Spiridoula Athanasiadou, Disease Systems, Animal and Veterinary Sciences, SRUC, Roslin Institute, Scotland, UK. The assay involved ligating 5 cm segments of the duodenum and maintaining 18-week-old Cobb and Hubbard broilers anaesthetized throughout the 4 h of the procedure [10]. The loops were washed with saline before injection and then injected with ~1 ml broth cultures containing 10X concentrated of *C. perfringens* CP1 (~2x10^8^cells/ml). *C. perfringens* CP1 cultures were grown anaerobically in 10 ml of TPG at 37 °C overnight, and the bacterial pellet was re-suspended in 1 ml of the culture supernatant and subsequently injected into the chicken loops. One loop per chicken was inoculated with sterile bacterial broth (TPG) as control. Following inoculations, the intestinal loops were replaced in the abdominal cavity and the abdominal wall and skin were stitched up to reduce losses of the body temperature. After 4 h of the inoculation, the intestinal loops were excised and the anaesthetized animals were then euthanized. Bacteria were then recovered from the loops by immediately mixing intestinal contents in 1 ml RNAlater buffer on ice and kept at 4 °C overnight. The contents of each loop were then maintained in RNAlater at −70 °C until RNA extraction. A segment of each loop was transferred to 10 % formalin for histological examination.

### Histopathological examination

Tissue was fixed in 10 % formalin and embedded in paraffin. Sections were cut and stained with hematoxylin and eosin (H&E). Stained sections were evaluated for lesions typical of microscopic NE lesions and determined to be either positive or negative.

### RNA isolation

For in vivo isolation, the intestinal contents in RNAlater were centrifuged at low speed to remove epithelial cells (5 min, 1000 x g). Thereafter, bacteria cells were pelleted by centrifugation (10 min, 10,000 x g). The pellet was resuspended in 1 ml of Trizol (Life Technologies), vortexed for 40 s and incubated at room temperature (RT) for 5 min. The Trizol suspension was transferred to frozen small tubes with Zirconia beads [11]. Bacterial cells were broken for 1 min using a MiniBeadBeater (BioSpec Products, Bartlesville, OK) at 6000 g, and then tubes were immediately placed on ice. Following this, 200 μL of chloroform was added to the solution, incubated at RT for 3 min and then spun down at 4 °C for 15 min at 12,000 g. The upper phase was collected and transferred to a fresh microcentrifuge tube before proceeding to RNA extraction using Direct-zol RNA MiniPrep columns (Zymo Research, Irvine, CA) following the manufacturer’s protocol.

For in vitro isolation, culture pellets of *C. perfringens* grown to OD_600_ 0.5 were resuspended in RNAlater to stabilize cellular RNA. The RNA was extracted using the hot-phenol method [12]. The pellets from each sample were resuspended in a total of 450 μl of RNase-free water before proceeding with RNA extraction by Zymo Direct-zol.

Total RNAs (from in vivo and in vitro experiments) were subjected to DNase I treatment (Ambion-Life Technologies, Burlington, ON) and PCR reactions were performed to check genomic DNA contamination of the total RNA samples. Bacterial ribosomal RNAs were removed with a Ribo-Zero Magnetic Kit (Gram-positive Bacteria) (Epicentre, Guelph, ON) and the enriched mRNA quality was assessed using a picochip 2100 Bioanalyzer. The RNA was stored at −80 °C and used for downstream molecular analysis.

### Whole transcript analysis by RNAseq

The enriched mRNA was converted to a stranded library using Illumina TruSeq RNA library prep protocol for the Illumina platform (Illumina Inc., San Diego, CA) by the Next-Generation Sequencing Facility, The Centre for Applied Genomics, MaRS Centre, Toronto, ON. The nine in vitro libraries (rich media [RM], poor media [PM], osmotic shock [OS]), including triplicates, were sequenced in one lane as a single pool, while the three in vivo [IV] libraries were pooled in another lane.

### RNAseq analysis

The processed reads from each sample were aligned using Bowtie2 version 2.1.0 against the corresponding *netB*-positive *C. perfringens* CP4 reference genome [4]. For differential expression analysis, raw read counts were imported and analyzed using the R/Bioconductor package edgeR version 3.2.4 [13].

Parameters for classifying significantly differentially expressed genes (DEGs) were ≥2-fold differences in the transcript abundance plus ≤1 % false discovery rate (FDR) [13].

To determine the functional annotation of DEGs, a BLAST (Basic Local Alignment Search Tool) alignment was performed by searching the Non-redundant (Nr) and Clusters of Orthologous Groups (COG) protein databases with an E-value ≤ 1e-5. The best matches were selected to annotate the DEGs. Finally, differentially expressed genes (DEGs) were then subjected to GO functional analysis using Blast2GO software4 (www.blast2go.com) utilizing default parameters, to annotate the DEGs’ major Gene Ontology (GO) categories, including molecular functions, biological processes, and cellular components [14]. GO analysis using the lists of differentially expressed genes revealed how they are collectively involved in a number of biological processes during infection or growth in a specific media. Enrichment of GO categories among DEGs was assessed by BinGO v2.4.4, a Cytoscape plugin (www.cytoscape.org). To cluster the samples based on the similarity of gene expression profiles unsupervised principal component analysis (PCA) and multi-dimensional scaling (MDS) were applied.

### Quantitative Real-Time PCR (qRT-PCR) validation

Four DEGs identified by RNA-seq were assayed by qRT-PCR. Purification of mRNA from in vitro and in vivo cultures was performed as mentioned above. Gene-specific primers used in this study are shown in Additional file 1: Table S1 (primers). For cDNA template synthesis, SuperScript III First-Strand Synthesis SuperMix (Invitrogen, Carlsbad, CA) with random hexamers was used following the manufacturer’s instructions. For qRT-PCR, LightCycler 480 SYBR Green I Master (Roche, Laval, QC) was used according to the manufacturer’s instructions, containing 0.5 μM each of the forward and reverse gene specific primers. Three biological replicates were analyzed per sample. The expression level of each sample was calculated using the 2^−ΔΔCt^ method, with the housekeeping gene 16SrRNA [15].

### Supporting data

The raw Illumina sequencing dataset of NE *C. perfringens* CP1 strain was submitted to the NCBI Gene expression Omnibus (GEO) under the accession #GSE79456.

## Results

### Chicken intestinal loops assay and histological lesions

**Fig. 1:**
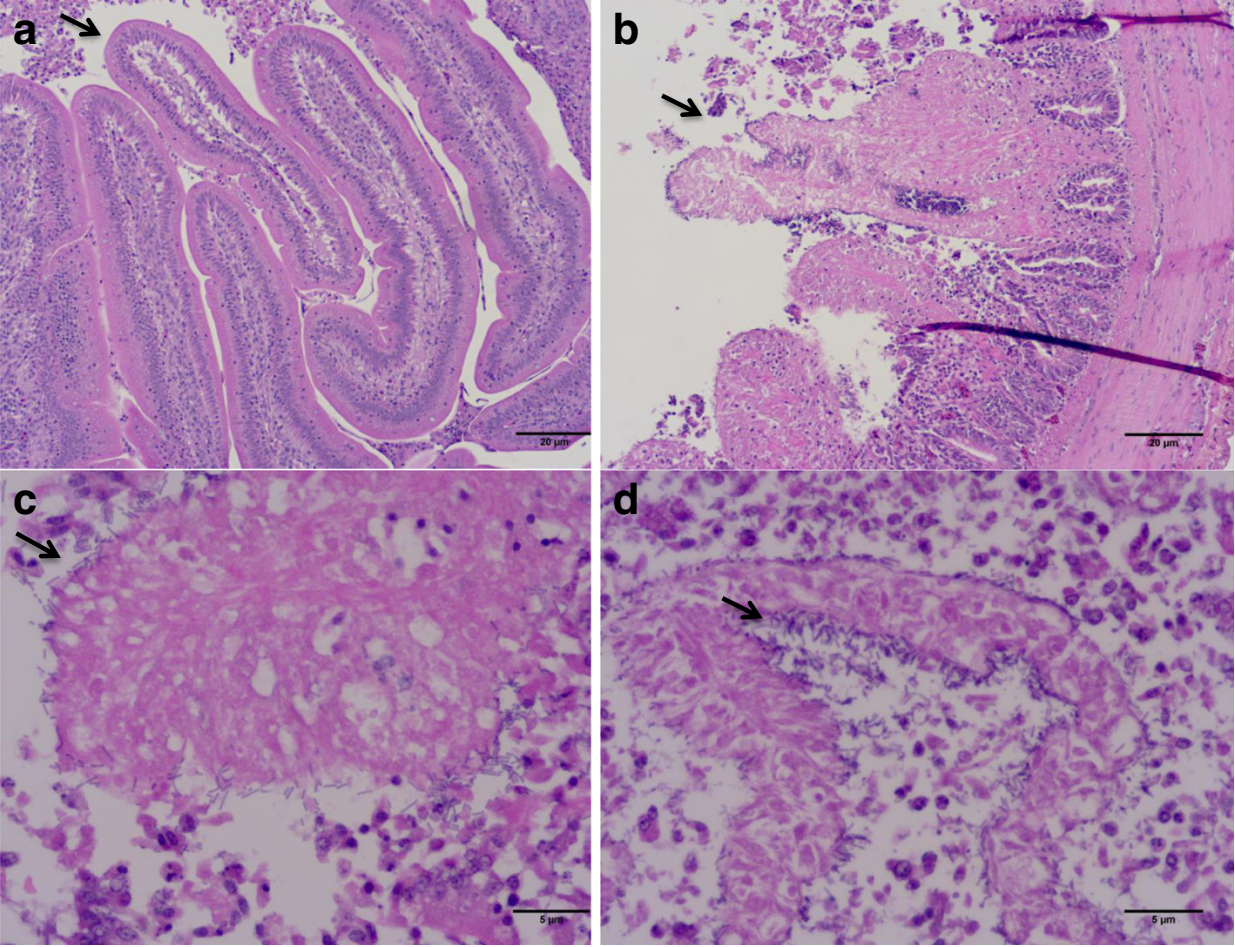
Histopathology of duodenum of broilers following chicken intestinal loops inoculated with *C. perfringens*. H&E-stained paraffin section of a duodenum villus. **a**: Enterocytes and villi appeared to be well organized in control loops with only TPG broth. **b**-**d**: Loops with *C. perfringens* inoculations, where the cell membranes have broken down and there is an appearance of coagulation necrosis. Large numbers of *C. perfringens* lining the damaged villi are visible in **c** and **d**. *Arrows point out C. perfringens*. Size bars/magnifications: **a** and **b** = 20 μm/X 100; **c** and **d** = 5 μm/X400

Intestinal distension resulting from accumulation of gas and fluid was observed 4 h post-infection in all the infected but not the TPG media control loops. Microscopic lesions typical of NE were detected in none of the control loops whereas lesions of coagulation necrosis similar to early NE were detected in all but one of the loops containing 10^9^ of the CP1 strain (Fig. 1). Some damage was evident in the control loops, which was likely due to the surgical procedure. Many *C. perfringens* could be seen lining the surface of the damaged intestinal epithelium of the infected loops as a marked feature of the infected intestinal sections (Fig. 1).

### Mapping and analysis of Illumina reads

Illumina HiSeq 2500 platform, paired-end and strand-specific sequencing produced an average read length of ~230 bp. The average number of reads obtained for in vitro samples was ~173 million reads per sample, with 99.9 % of reads mapping to the *C. perfringens* CP4 strain genome. The number of reads for in vivo samples was on average ~544.4 million reads, with 6.4 % mapped to CP4 genome. In general, out of 3573 genes, 2287 (64 %) showed transcription.

**Fig. 2:**
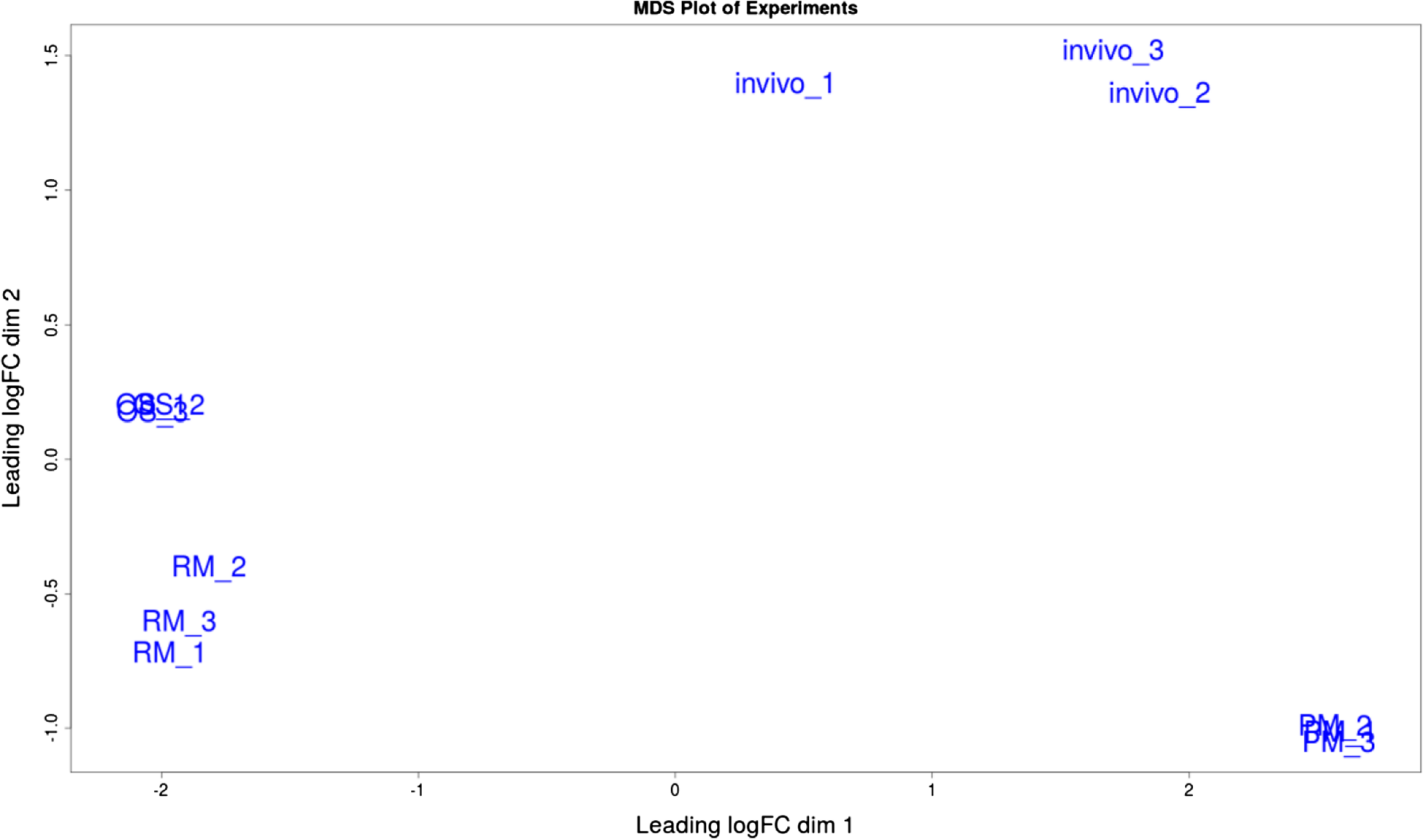
Multidimensional scaling (MDS) plot. Distance between sample labels indicates similarity of samples and the experiments

The Multi-Dimensional Scaling (MDS) plot (Fig. 2) using output data from edgeR, indicated that the biological replicates of the *C. perfringens* CP1 strain collected during in vitro (RM, PM, OS) and in vivo growths were clustered together. There was more variability between the different experimental conditions than within each biological replicate group. The in vivo samples appeared more heterogeneous than the RM and OS samples, suggesting a treatment effect but all different experimental growth conditions were functionally distinct.

### Differentially-expressed genes (DEGs) of in vivo versus in vitro samples

Selection of genes significantly differentially expressed were based on cut-off values of ≥2-fold, FDR ≤ 1 %, p ≤ 0.05 of in vivo compared to in vitro conditions. Analysis of DEGs were subdivided in three categories: (i) Genes induced in vivo (during chicken infection) (ii) Genes induced under in vitro conditions; (iii) Virulence and virulence-associated genes. Volcano graphics were used to visualize the differentially expressed genes in different growth conditions (Additional file 2: Figure S1).

### Genes induced in vivo

The number of differentially expressed genes (DEGs) during chicken intestinal loop infection is listed in Table 1. Comparison of in vivo samples to RM (in vitro) samples identified 673 genes that were significantly differentially expressed (Table 1 and Additional file 3: Table S2). The results showed that 387 (57.5 %) genes were upregulated (increased numbers of transcripts) and 286 (42.5 %) genes were downregulated. In comparing samples from in vivo to PM (in vitro), a total of 382 genes were significantly differentially expressed, with a majority (234 genes, 58.9 %) displaying significant reductions in expression compared to those (148 genes, 38.7 %) that had increased expression (Table 1). The final analysis of in vivo samples compared to OS (in vitro) showed that 521 genes were significantly differentially expressed with, the majority (376, 72 %) being upregulated compared to those (145, 27.8 %) that were downregulated.

**Table 1:** The number of significantly differentially expressed genes (DEGs) in NE-strain CP1 under different growth conditions

Growth Conditions^1^		edgeR^2^ number of genes	UpRegulated^3^	DownRegulated^3^
in vivo	IV x RM	673	387	286
	IV x PM	382	148	234
	IV x OS	521	376	145
in vitro	PM x RM	600	441	159
	OS x RM	196	44	152

^1^ *RM:* rich medium, *PM:* poor medium, *OS:* osmotic shock

^2^egdeR: *log*FC: ≥2-fold differences in the transcript abundance plus ≤1 % false discovery rate (FDR)

^3^Number of genes up- or downregulated

**Fig. 3:**
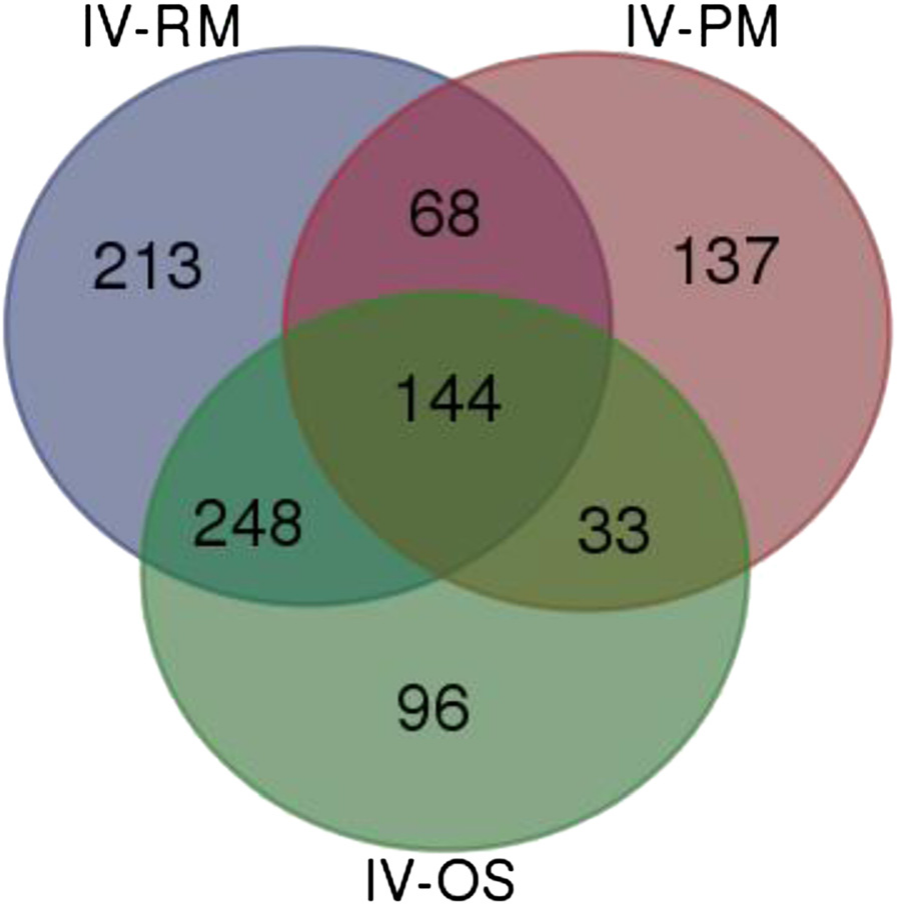
Venn diagram comparing differentially expressed genes (DEGs) among in vivo vs in vitro conditions (IV-RM, IV-PM, IV-OS). Significantly DEGs, with *log *ratio ≥ 2.0 and FDR- adjusted *p* <0.05, of in vivo (IV), Poor Media (PM), Rich Media (RM) and Osmotic Shock (OS). (bioinformatics.psb.ugent.be/webtools/Venn/)

**Fig. 4:**
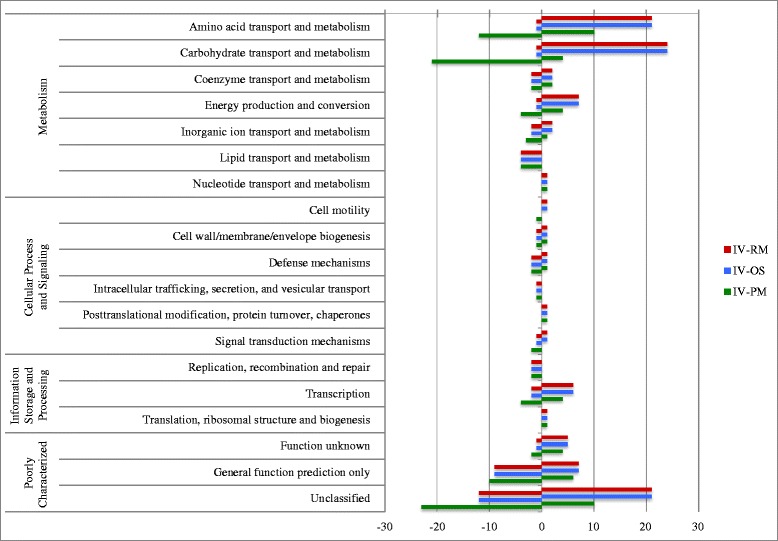
Histogram of clusters of orthologous groups (COGs) of 144 common DEGs of in vivo growth conditions. *red*: IV-RM, *blue*: IV-OS, *green*: IV-PM, genes are represented up-and downregulated. *x*-axis represents COG protein groups and *y*-axis represents the number of genes –negative: downregulated and +: upregulated

A Venn diagram (Fig. 3) was used to show the specific DEGs altered by the 3 in vivo growth conditions only. A group of 144 genes were identified as significantly differentially expressed in all comparisons between the in vivo conditions and each of the in vitro conditions. The analysis of growth conditions IV vs RM and IV vs OS had the same transcription patterns with 102 genes upregulated and 42 genes downregulated, while IV vs PM showed some differences, with 50 genes upregulated and 94 genes downregulated, which suggests that chicken intestine environment represents more limiting nutritional conditions than the in vitro poor media. To better understand the significance of the common 144 differentially regulated genes in each of the in vivo comparisons, they were assigned to their respective COG (Clusters of Orthologous Groups) of proteins (Fig. 4). The majority of transcriptional changes were seen in the Metabolism COG class (47 %), specifically amino acid and carbohydrate transport, followed by 11 (7.6 %) genes in the Information Storage and Processing class, and 10 (6.9 %) genes in the Cellular Process and Signaling, and finally, 55 genes (38.2 %) in the Poorly Characterized class. In the Metabolism class, 57 genes (39.6 %) out of 144 were upregulated suggesting an activated metabolic state.

### Genes induced in vitro conditions

A large number of DEGs was found when comparing RM vs PM, with 600 genes in total of which 441 (73.5 %) were upregulated. The lowest number of DEGs was detected in the comparison of the RM to OS (196 genes), of which 152 (77.5 %) were downregulated (Table 1).

### Virulence and virulence-associated genes

A set of 92 genes related to virulence, including genes in the pathogenicity loci (NELoc-1, - 2, -3), genes encoding putative adhesins (VR10B), and VirR/VirS regulated virulence factors (chromosomal and plasmid toxins and enzymes) and regulatory proteins (quorum-sensing, and putative regulators), representing the netB-*positive C. perfringens* named as the virulome were grouped for analysis (Additional file 4: Table S3). Table 2 shows genes in this grouping with significant differential expression. Out of the 92 genes in the virulome, 27 genes in the IV vs RM comparisons were significantly differentially expressed (17 downregulated, 10 upregulated), whereas virulome genes significantly differentially expressed in the IV vs PM and in the IV vs OS comparisons were 8 genes (5 downregulated, 3 upregulated) and 22 genes (13 upregulated, 9 downregulated), respectively. The comparisons of the in vitro conditions, the PM vs RM comparison showed 21 DEGs (13 downregulated, 8 upregulated) and in the OS vs RM comparison just 8 DEGs (7 downregulated, 1 upregulated).

**Table 2:** Virulence and virulence-associated genes in *C. perfringens* strain CP1 that were significantly expressed under different conditions

			in vivo-RM			in vivo-PM			in vivo-OS			PM-RM			OS-RM		
Feature	Locus Tag	Predict product (gene name)	Fold	*log*2FC	FDR	Fold	*log*2FC	FDR	Fold	*log*2FC	FDR	Fold	*log*2FC	FDR	Fold	*log*2FC	FDR
V	CP4_0080	phospholipase C (plc)	9.3	3.2	0.0				7.1	2.8	0.0	6.9	2.8	0.0			
V	CP4_0193	peptidoglycan protein (cna)	−8.5	−3.1	0.0										−7.5	−2.9	0.0
V	CP4_0211	collagenase_kappa toxin (colA)	4.7	2.2	0.0												
V	CP4_0212	collagenase_kappa toxin (colA)	4.9	2.3	0.0												
QS	CP4_0214	UbiE/COQ5 family methlytransferase (ycgJ)	−59.4	−5.9	0.0							−22.5	−4.5	0.0	−112.0	−6.8	0.0
QS	CP4_0215	cystathionine beta-lyase (metB)	−59.3	−5.9	0.0							−18.6	−4.2	0.0	−40.2	−5.3	0.0
QS	CP4_0216	cysteine synthase (cysK)	−30.7	−4.9	0.0							−17.8	−4.2	0.0	−31.5	−5.0	0.0
QS	CP4_0217	S-ribosylhomocysteinase (luxS)	−27.3	−4.8	0.0							−7.2	−2.9	0.0	−29.8	−4.9	0.0
NELoc-2	CP4_0460	putative VTC domain superfamily			0.0				−5.8	−2.5	0.0						
NELoc-2	CP4_0461	putative tubulin/FtsZ, GTPase	−6.3	−2.7	0.0	−5.4	−2.4	0.0	−8.3	−3.1	0.0						
VR10B	CP4_0572	collagen adhesin (cnaA)			0.0				−7.4	−2.9	0.0						
VR10B	CP4_0573	signal peptidase I (lepB)	−5.3	−2.4	0.0				−11.0	−3.5	0.0	−4.2	−2.1	0.0			
VR10B	CP4_0574	PitB	−4.6	−2.2	0.0				−9.2	−3.2	0.0						
VR10B	CP4_0575	sortase, SrtB family (srtB)	−4.1	−2.0	0.0				−8.7	−3.1	0.0	−4.3	−2.1	0.0			
VR10B	CP4_0576	putative streptococcal pilin isopeptide link	−4.2	−2.1	0.0				−9.8	−3.3	0.0						
V	CP4_0806	sialidase (nanI)	76.1	6.3	0.0				129.2	7.0	0.0	132.8	7.1	0.0			
regulator	CP4_0922	VirT (virT)	30.1	4.9	0.0				32.4	5.0	0.0	78.1	6.3	0.0			
regulator	CP4_1328	VR-RNA (vrr)			0.0				−16.1	−4.0	0.0	−5.6	−2.5	0.0	4.2	2.1	0.0
V	CP4_1570	enterotoxin (entD)	4.0	2.0	0.0												
V	CP4_1581	hyaluronidase _mu-toxin (nagJ)	4.9	2.3	0.0				13.0	3.7	0.0	5.0	2.3	0.0			
V	CP4_2582	hyaluronidase _mu-toxin (nagA)			0.0							8.2	3.0	0.0			
plasmid	CP4_3441	beta2 toxin (cpb2)							8.3	3.1	0.0	6.9	2.8	0.0			
NELoc-1	CP4_3443	beta-lactamase domain-containing protein			0.3	6.7	2.7	0.0				−4.9	−2.3	0.0			
NELoc-1	CP4_3444	M protein trans-regulator	−15.7	−4.0	0.0	−4.6	−2.3	0.3	−24.3	−4.6	0.0						
NELoc-1	CP4_3445	SAM domain-containing protein	−14.0	−3.8	0.0	−22.7	−4.5	0.0	−14.4	−3.9	0.0						
NELoc-1	CP4_3446	putative internalin	−8.5	−3.1	0.1	−8.0	−3.0	0.1	−20.2	−4.3	0.0						
NELoc-1	CP4_3454	chitinase B	19.6	4.3	0.0				70.3	6.1	0.0	26.9	4.8	0.0			
NELoc-1	CP4_3455	chitodextrinase	31.1	5.0	0.0				112.2	6.8	0.0	39.2	5.3	0.0			
NELoc-1	CP4_3458	pore-forming toxin_NetB (part)			0.0	28.2	4.8	0.0				−8.5	−3.1	0.0			
NELoc-1	CP4_3459	pore-forming toxin_NetB (part)	4.2	2.1	0.0	29.9	4.9	0.0	4.5	2.2	0.0	−7.1	−2.8	0.0			
NELoc-1	CP4_3460	hypothetical protein	−12.9	−3.7	0.0										−4.5	−2.2	0.0
NELoc-1	CP4_3461	hypothetical protein	−4.2	−2.1	0.0				−6.1	−2.6	0.0						
NELoc-1	CP4_3464	resolvase/recombinase			0.0							−7.7	−3.0	0.0			
NELoc-1	CP4_3465	resolvase/recombinase			0.0							−7.5	−2.9	0.0			
NELoc-1	CP4_3475	ABC transporter			0.0							−5.7	−2.5	0.0			
NELoc-1	CP4_3478	diguanylate cyclase/phosphodiesterase	−4.1	−2.0	0.0	−4.9	−2.3	0.0	−6.5	−2.7	0.0						
NELoc-3	CP4_3569	resolvase/recombinase							5.8	2.5	0.0						
NELoc-3	CP4_3570	hypothetical protein	−5.8	−2.5	0.1										−4.4	−2.2	0.0

^1^Features: V: Virulence factors; QS: Quorum-sensing; VR10B: Variable region; NELoc-1, 2, 3: Pathogenicity locus 1, 2, 3

^2^ Locus Tag referent to CP4 genome

^3^Differential expression (DE) calculated by edgeR method, significantly expressed genes (fold change ≥ 2 and FDR = false discovery rate *p*-value ≤ 0.05)

The quorum sensing gene *luxS* (CP4_0217), which plays an important role in the regulation of virulence factors, as well as *ycgJ, metB and cysK* genes (part of the *luxS* operon) (CP4_0214, CP4_0215, CP4_0216), were all significantly downregulated in three of the growth conditions, including IV vs RM (−27-fold), PM vs RM (−7-fold) and OS vs RM (−29-fold) (Table 2). The VR-RNA gene, a small RNA molecule that is part of VirR/VirS system, was only significantly increased under OS vs RM (4-fold change) growth conditions, but was significantly decreased under other conditions. Interestingly, the RNA regulator, VirT, was significantly upregulated, it was increased 30-fold in the IV-RM, 32-fold in the IV-OS and 78-fold under in vitro nutrient deprivation (PM-RM) (Table 2).

**Fig. 5:**
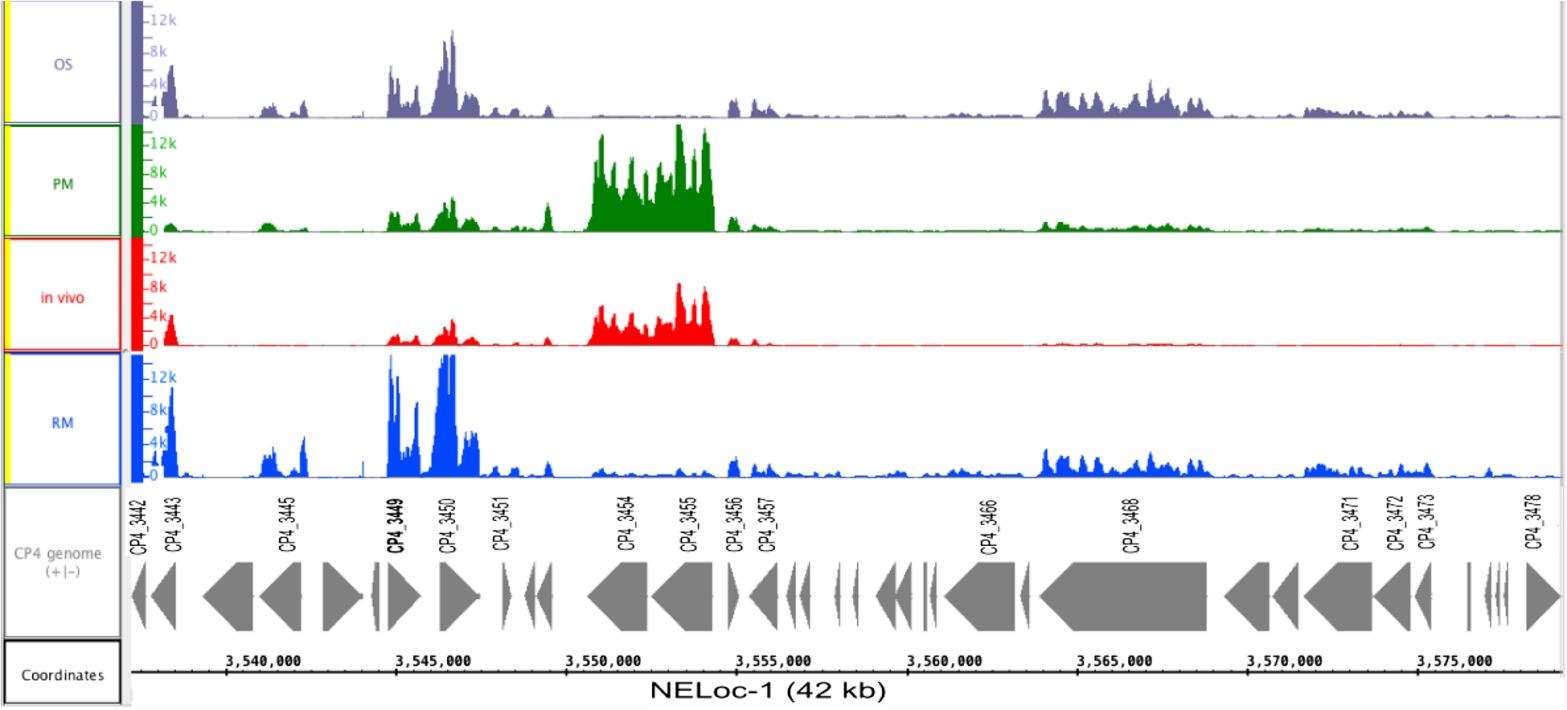
Visualization of Mapped Sequence Reads for the NELoc-1. The genetic organization of NELoc-1 is shown, each *arrow* representing a predicted gene. Predicted functional annotations and locus tags are shown above each gene, respectively. Mapped sequence reads for the NELoc1 genes were shown in the IGB browser. The figure shows the expression of CP4_3443 (β-lactamase protein), CP4_3449 (NetB), CP4_3450 (ricin-protein), CP4_3454 and CP4_3455 (chitinase A and chitinase B), CP4_3468 (F5/8 type C protein) expressed in different growth conditions: OS: Osmotic Shock, PM: Poor media, in vivo: from chicken intestinal loops, RM: rich media

Out of 37 genes present on NELoc-1, which contains *netB,* only 14 genes showed differential expression (Table 2, Fig. 5). *netB*, a critically-important virulence-associated gene in the NELoc1, although expressed as shown in Fig. 5, was not significantly differentially expressed when compared among different growth conditions. The expression of two chitinase genes (CP4_3454, CP4_3455) was significantly upregulated in most of the in vivo growth analyses (increased from 19-fold to 112-fold), whereas three genes that encode a M protein trans-regulator (CP4_3444), a putative radical SAM domain-protein (CP4_3445) and a putative internalin gene (CP4_3446) were significantly down-regulated in all in vivo comparisons (Table 2). The potential c-di-GMP signaling system encoded by the *dgc* gene (CP4_3478) was also significantly downregulated from −2 to −6-fold changes in all in vivo (IV) comparisons (Table 2). In the pathogenicity locus NELoc-2, two genes (CP4_0460 and CP4_0461) consistently showed a significant decrease of transcripts in all in vivo comparisons (from −2 to −8-fold). Only two genes of NELoc-3 (CP4_3569 and CP4_3570) were significantly differentially expressed, whereas the hypothetical protein (CP4_3570) was downregulated 5.8- fold and 4.4-fold changes in IV vs RM and OS vs RM, respectively. The resolvase (CP4_3569) was upregulated 5.8-fold in IV vs OS comparison.

Transcription of genes encoding putative adhesion factors (CP4_0572, CP4_0573, CP4_0574, CP4_0575, CP4_0576), located in the VR-10B region exclusively found in poultry isolates [16], were mostly repressed, decreasing expression by 4-to 11-fold changes (Table 2). In marked contrast to the general downregulation of most of the virulome, in vivo in comparison to RM growth showed significantly increased expression of the toxins such as phospholipase C (α-toxin) (CP4_0080) to 9-fold, k toxins (CP4_0211, CP4_0212) around 4-fold, whereas the plasmid-encoded β-2 toxin (CP4_3441) increased by 8-fold and ~7-fold changes in IV vs OS and PM vs RM, respectively. Collagen-binding surface proteins (CP4_0193, CP4_0572) were significantly downregulated in IV vs RM, OS vs RM and IV vs OS growth conditions by approximately a 7-fold change (Table 2). By contrast, the major sialidase gene (*nanI*) (CP4_0806) was markedly upregulated by 76- fold, 129-fold and 132-fold changes under IV vs RM, IV vs OS and PM vs RM, respectively.

In the *C. perfringens* CP4 genome sequence [4], 52 genes were classified as two-component signal systems (TCS), including 30 sensor histidine kinases and 22 response regulators (Additional file 5: Table S4). The VirR/VirS and *agrR*, key regulators of *C. perfringens* virulence, were not significantly expressed, and were slightly repressed under most growth conditions. The AraC TCS family (CP4_1221-CP4_1222) was the most significantly upregulated system, with 11- and 6.4-fold changes in IV vs RM, 8-fold change IV vs OS, and 15- and 5-folds in RM vs PM. Under the same conditions, the *citA*-*citB* TCS (CP4_0590-CP4_0591) was the most downregulated. The orphan response toxin gene regulator RevR (CP4_0707) showed significantly upregulation (7-fold change) only in vivo as compared to in vitro PM growth. However, the genes under RevR control, such as α-clostripain (*closS1*) and the hyaluronidase genes (*nag*H and *nag*L) were not significantly differentially expressed under any conditions in this study (Additional file 5: Table S4).

**Fig. 6:**
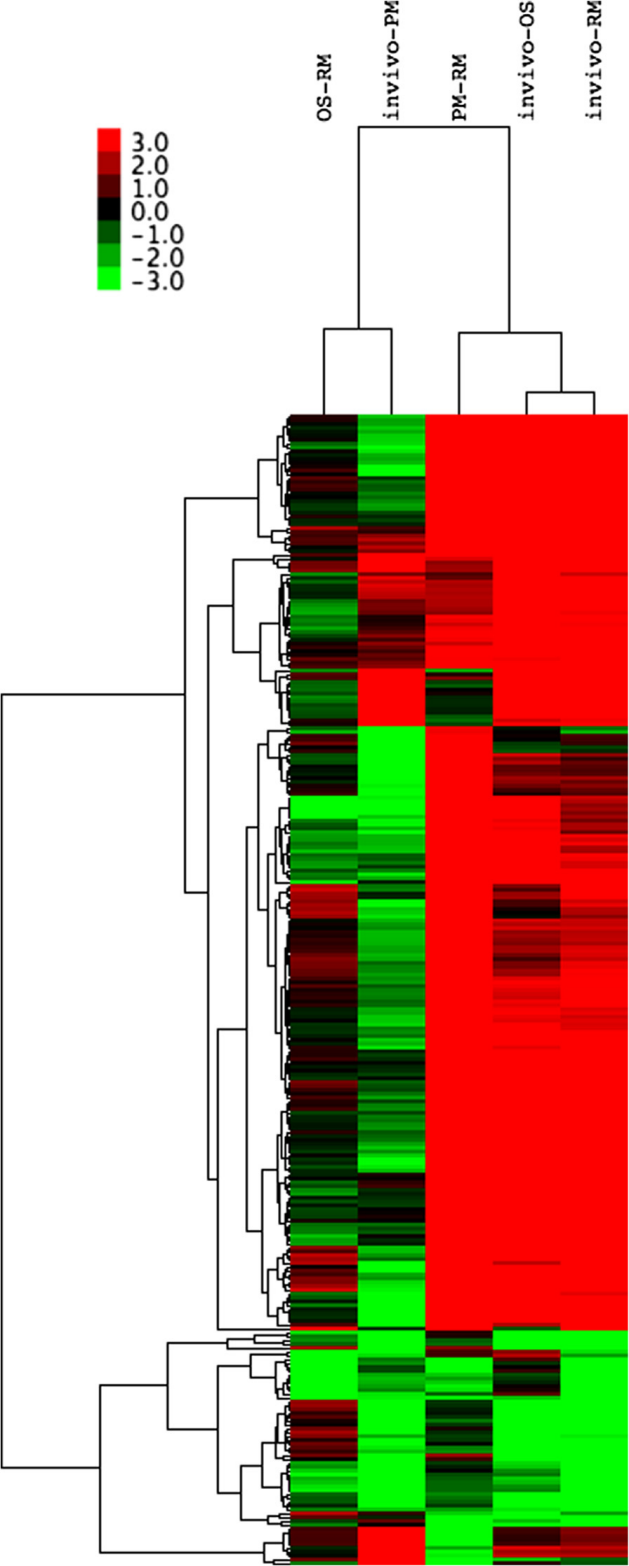
Hierarchical clustering in heat map format of all DEGs in NE *C. perfringens* strain CP1 grown under different conditions. Each horizontal row represents a differentially expressed gene, whereas each column represents a different growth condition. Green represents downregulated expression and red represents upregulated expression. Log_2_ values were used to cluster all the DEGs in Java TreeView by hierarchical clustering using Euclidean distance and pairwise average linkage methods

Global DEGs patterns occurring in the experimental conditions are shown in the hierarchical heat map (Fig. 6). It is clear that the heat map shows distinct expression profiles in response to growth conditions. These different patterns related to gene expression cluster depending on growth conditions; thus IV vs RM shows similarity with PM vs RM and IV vs OS. By contrast, IV vs PM and OS vs RM are not similar to the other conditions. IV vs PM shows considerable downregulation compared to other IV comparisons.

**Fig. 7:**
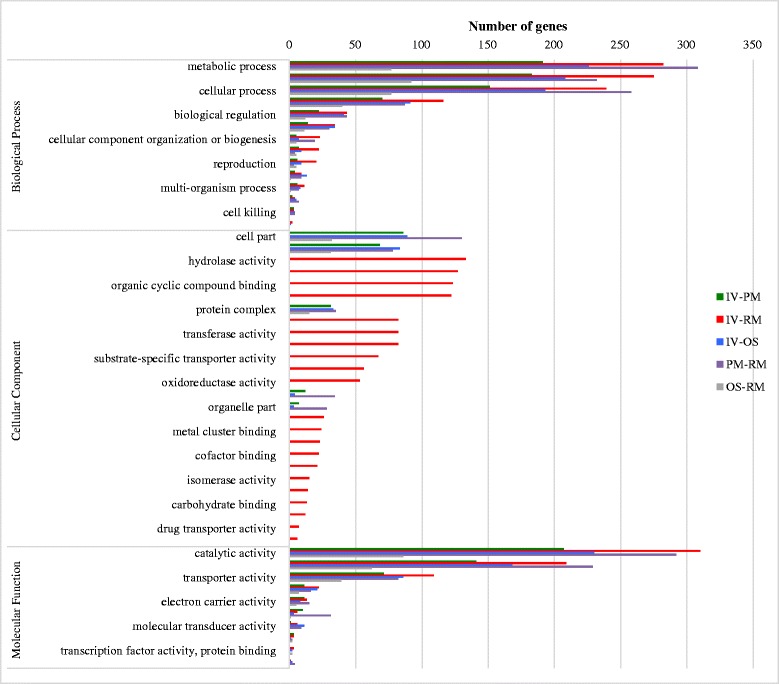
Functional analysis of all DEGs of NE *C. perfringens* CP1 genes. The graph shows GO terms significantly associated with genes that show significant enrichment in different growths. Bars correspond to number of genes associated with each GO term on Biological Process, Cellular Component and Molecular Function

Based on volcano plot analysis (Additional file 2: Figure S1) and comparison of number of DEGs changed by different growth factors (Fig. 7, Table 1), the IV vs RM comparison had the greatest effect on transcription followed by OS and then growth in PM.

### Functional analysis and classification of DEGs

To better understand the transcriptome of NE *C. perfringens*, GO (Gene Ontology) was applied to classify functions of the predicted genes.

From the NE *C. perfringens* CP4 genome, 2282 genes were grouped to at least one GO term and were classified into three functional categories (cellular component, biological process and molecular function). To appreciate the putative function of the all differentially expressed genes (DEGs) for in vivo (IV-PM, IV-RM, IV-OS) and in vitro (PM-RM, OS-RM) groups, we used GO enrichment, which provides statistical support in GO terms for differentially expressed and over-represented genes. In general, the enrichment analyses of DEGs showed that all comparison done with the in vivo and in vitro growth conditions belonged mainly to two categories: biological processes and molecular functions (Fig. 7, Additional file 6: Table S5). The total DEGs of IV vs RM (673), IV vs PM (382), IV vs OS (521) and PM vs RM (600) groups were identified and found to be over-represented in three different GO terms: biological process (BP), molecular function (MF) and cellular component (CC) (Additional file 6: Table S5). Among them, the majority of DEGs of all groups (IV-PM, IV-RM, OS-RM and PM-RM) but not OS vs RM were found to be involved in the organic substance metabolic processes (GO:0071704), cellular metabolic processes (GO:0044237), and single-organism metabolic processes (GO:0044710) whereas the IV-OS showed DEGs distributed in the organic cyclic compound binding (GO:0097159), heterocyclic compound binding (GO:1901363), and ion binding categories (GO:0043167).

Specifically, the two related groups PM-RM and IV-RM showed DEGs over-represented in ethanolamine metabolic process (GO:0006580-BP), structural constituent of ribosome (GO:0003735-MF), organellar large ribosomal subunit (GO:0000315-CC), rRNA binding (GO:0019843-MF), carbohydrate binding (GO:0030246-MF) and ‘de novo’ IMP biosynthetic process (GO:0006189), and ATP-binding cassette (ABC) transporter complex (GO:0043190), respectively.

### Validation of RNASeq data by qRT-PCR

qRT-PCR was performed on four DEGs to confirm the data generated by RNAseq in vivo (IV) compared to in vitro samples (RM, PM, OS). The genes selected to be amplified by qRT-PCR were *virT* (CP4_0922), *pts* (phosphotransferase system mannitol/fructose-specific IIA domain - CP4_0736), *plc* (α-toxin -CP4_0080) and *luxS* (CP4_0217). The direction of fold change of these selected genes using qRT-PCR was in agreement with RNAseq (Additional file 1: Table S1 (qRTPCR vs RNASeq)), except for two samples (PM vs RM and OS vs RM) for *luxS* and *virT*, respectively. Although two genes had different directions of fold change, the remaining 18 gene comparisons had similar expression patterns. Therefore the qRT-PCR results confirm the accuracy and consistency of the RNAseq data.

## Discussion

This study, which analysed the entire *C. perfringens* genome using the ssRNAseq technique for the first time, was designed to identify genes that were differentially expressed *in vivo* and *in vitro* in a chicken NE-associated, *netB*-positive, isolate of *C. perfringens*. Chicken intestinal loops were used to represent in vivo infection in a single time point (4 h) infected under controlled conditions and the in vitro growth conditions involved addition to different media (increased osmolarity, nutritional limitation, fresh rich medium) to represent aspects of the different environments that might be encountered by *C. perfringens* during the pathogenesis of necrotic enteritis.

Intestinal loops showed histopathological effects similar to early characteristic NE [17] during bacterial colonization (4 h), but the majority of virulence and virulence-associated genes were either repressed or lower expressed, suggesting that the damage observed might be produced by toxins in the broth supernatant.

The sequencing data analyses obtained clearly showed a significant impact on gene expression of the *C. perfringens* transcriptome during intestinal infection and under different conditions in vitro. The overall data in the MDS plot (Fig. 2) highlighted the difference between the samples and showed the greatest variance in the in vivo samples; this latter was not unexpected since obtaining and processing the in vivo samples was a relatively complex process.

The majority of the total of 144 genes that responded to all the in vivo comparisons (Fig. 3), 68 genes (47.2 %) (Fig. 4), were related to the COG groups of amino acid and carbohydrate transport (Metabolism class) indicating increased source and transport of amino acids/carbohydrates, which suggests that *C. perfringens* was adapting its metabolism to diverse environments, as is important during infection.

A distinct hierarchical cluster profile was observed in the heat map of gene expression (Fig. 6) where it is clear that IV vs RM, PM vs RM and IV vs OS groups cluster together due to similar gene expression patterns. The combination of heat map results, which shows up- and downregulated genes, and general analysis of volcano plots (Additional file 2: Figure S1), support the conclusion that the gene expression patterns were related to nutritional deficiency (in vivo and PM) and increased osmotic pressure (OS) that occurred in the environment of the chicken intestine and under in vitro conditions. It is noteworthy that DEG in vivo showed greatest similarities to those of bacterial growth in PM, evident in the heat map (Fig. 6), where PM vs RM cluster together with IV vs RM and IV vs OS. The comparison IV vs PM showed that the majority of genes were downregulated, suggesting that there was similar or greater nutritional deficiency in the intestinal environment. It is interesting to note from the heat map analysis the relatively fast (4 h in vivo and 1 h in vitro) up- and downregulation of a similar group of genes under very different conditions, which demonstrates a promptly organized transcriptional regulation during infection that did not involve the well-known regulators Agr and VirR/VirS.

Within the time of observation, 673 genes (19.4 % genome) showed differential expression in 4 h in the IV vs RM group and 600 genes (17.3 % genome) in 1 h in the PM vs RM group (Table 1). Most DEGs of the comparison groups IV vs RM, IV vs OS and PM vs RM (Fig. 7) were localized to metabolic processes and cellular processes, as well as catalytic activity and transporter activities, suggesting their involvement in the NE *C. perfringens* adaptation to the intestinal environment and to a nutrient poor environment, an adaptation similar to that reported for *C. difficile* [18]. These include components of ethanolamine metabolic processing and ATP-binding cassette (ABC) transporter complex, also described to be important for adaptation to environment [19, 20].

The pathogenesis of NE is complex [17] and is likely to involve many genes on the pathogenicity loci [4], chromosomal genes associated with NE strains [16] and other genes controlled by the VirRS regulatory system. In *C. perfringens*, the two-component regulatory system VirR/VirS and regulatory RNA (VR-RNA) cascade have major control of virulence genes, as well genes for enzymes involved in energy production, acquisition of nutrient and other cellular functions [21]. Two crucial quorum-sensing (QS) systems, a cell-to-cell communication using chemical signals to monitor bacterial environment and regulate many bacterial genes, such as Agr-system and LuxS are important for the regulation of toxin genes since they regulate VirR/VirS. The QS system switches on the VirR/VirS two-component regulatory system, and initiates the disease process [22, 23]. Analysis of the behaviour of these major regulatory genes (Additional file 5: Table S4), including either the VirR/VirS genes or Agr gene, showed no significant expression under any conditions, while genes of the LuxS operon were all significantly downregulated under all growth conditions. The impact of downregulation or no significant change in the expression of the major regulatory genes was clearly evident in the repression/lack of expression of most of the virulence-associated genes (Table 2, Additional file 4: Table S3, Fig. 5). It is interesting that the response to environmental cues led to the repression of *netB*, which may be directly linked to VirR/VirS repression since *netB* is under the control of the VirR/VirS two-component regulatory system [23, 24]. Therefore these results are in agreement with the conclusion that most of the virulence-associated genes in *C. perfringen*s are controlled by Agr and VirR/VirS [25, 26]. Taken together, the results from in vivo and in vitro growth conditions and heat map cluster observations suggest that unknown regulatory elements act to induce/repress a large group of genes, as well their importance in the initial steps of colonization.

Adherence of enteropathogens to intestinal epithelium cells is an important first step in establishing an infection [27]. Degradation of the intestinal mucus barrier and colonization of this site is likely to be the critical step in the initiation of NE [28]. Interestingly, the chitinase gene (CP4_3444) and the chitin-binding protein (CBP) (CP4-3445) on the pathogenicity locus, NELoc-1, were markedly upregulated (from 20-fold to 110-fold) (Table 2, Fig. 5). These chitinases belong to the glycoside hydrolase (GH) family 18 that is widespread among bacterial pathogens [29]. These glycosyl hydrolases cleave the glycosidic bonds of glycans and are increasingly being recognized as virulence-associated factors [29]. They are common in bacterial pathogens involved in mucus colonization and degradation, which is very likely an initial step in the pathogenesis of NE [17, 29]. The upregulation of these two chitinase genes was in marked contrast to the downregulation or lack of change of the rest of NELoc-1. These findings suggest either that the *C. perfringens* may have been in the early stages of a response to its presence in the intestine by starting to upregulate virulence-related genes or that the transition from a nutritionally rich to a poor medium, which also induced transcription of these two genes (Table 2), is a signal for chitinase gene upregulation, or both.

Degradation of mucus in the small intestine is likely a critical source of nutrients for *C. perfringens* [30, 31]. This allows the bacterium to form localized microcolonies on the mucosal surface and to produce the QS auto-inducing peptides that start the VirR/VirS cascade [23]. The demonstration that the most upregulated gene in the virulome group was the prominent sialidase (*nanI*) (up to 130-fold) (Table 2) also supports the conclusion that the transcriptomic changes observed reflected changes expected in the initial stage of intestinal colonization. *C. perfringens* utilizes sialic acid found in mucin as a nutrient source through the action of sialidases, which appear to be involved in promoting adhesion of *C. perfringens* through modification of epithelial cell surfaces to allow binding to sialidase-exposed receptors on enterocyte cell surfaces [17, 32, 33]. It is also noteworthy that the increased transcription of *nanI* that occurred in vivo was also observed in the transition from RM to PM (Table 2).

Interestingly, some toxin genes, notably α-toxin gene (*cpa*), kappa toxin genes (*colA*), hyaluronidase (*nag*A), hyaluronidase (*nag*J) and β-2 toxin (*cpb2*) were significantly upregulated under most growth conditions (Table 2). Despite the dramatic repression of Agr and VirR/VirS, the transcription of α-toxin (*cpa*) and β2-toxin (*cpb2*) was significantly increased, suggesting that these regulators may not be critical for the regulation of α- and β2 toxins, dissimilar to what has been previously reported [23, 34]. This group of toxins thus seems to be partly independent of VirR/VirS and may be involved as part of early colonization steps.

The small RNA, VR-RNA system by itself controls at least 147 genes including *plc*, 15 collagenases (*cna*), siliadase (*nanJ*), sialidase (*nanI*), hyaluronidase (*nagL*) and many other virulence-related and housekeeping genes [22]. The notable exception to the downregulation of VR-RNA and most genes of the virulome was the upregulation of one collagenase gene (*cna*) and particularly the sialidase (*nanI*) discussed earlier. The VR-RNA was only significantly upregulated in rich media (RM) compared to osmotic shock (OS), but *cna, nanJ* and *nanI* were not significantly transcribed (Additional file 4: Table S3). This suggests that the VR-RNA acts a repressor of these genes.

*virT* (RNA regulator) has been suggested to have a role of fine-tuning the transcription of VirR/VirS-regulated genes; it negatively regulates *pfoA* transcription [23]. Our results support these findings since *vir*T was significantly upregulated (30-, 32-, 78-fold changes) under three growth conditions (IV-RM, IV-OS and PM-RM) whereas *pfoA* did not show any expression.

The regulatory network in *C. perfringens* is very complex and likely has unknown regulator(s) for both chromosomally and plasmid encoded toxin genes*.* The classical two-component systems (TCS) are composed of a histidine kinase (HK) that responds to specific stimuli, and a response regulator (RR) involved in regulating gene expression [35]. In 2011, Dintner *et al.* described a widespread distribution of unique and self-sufficient detoxification modules against antimicrobial peptides (AP), whereby ABC- type of transporters with unusual domain architecture were controlled by an adjacent TCS. This detoxification module consists of a “three-component system” (3CS) (a putative periplasmic ABC transporter substrate-binding protein and a two-component system). We observed that three out of the six TCS (CP4_0635–0637; CP4_1221-1223; CP4_2491-2493) (Additional file 5: Table S4) that were significantly upregulated in different growth conditions (IV-RM, IV-OS, PM-RM) have an adjacent periplasmic ABC-transporter with these features, which were also significantly upregulated. The 3 CS “CP4_0635-0637” and “CP4_1221-1223” comprises the periplasmic-binding component of ABC transport systems specific for trehalose/maltose and a two-component system of the AraC family. Finally, “CP4_2491-2493” also includes a periplasmic-binding component of ABC transport systems specific for a molybdate-binding protein and a two-component system of the AraC family. These periplasmic ABC transporters act as a single transmembrane sensor to assist TCSs to respond to extracellular conditions [19]. To survive during colonization or infection *C. perfringens* might avoid these innate host defenses that form such a key component of mucus, using these 3CS as defense against AP [36].

Interestingly, TCS “CP4_0982-0983” (ethanolamine sensory transduction histidine kinase) which was significantly upregulated in PM-RM (6-fold) is adjacent to the ethanolamine operon that was also significantly upregulated (Additional file 5: Table S4). Ethanolamine, a breakdown product of the membrane lipid phosphatidylethanolamine, is prevalent in the gastrointestinal environment [20]. There is a global association of ethanolamine degradation with intestinal bacterial pathogens [20]. For example, three intestinal pathogenic bacteria (*Salmonella enterica*, *C. perfringens*, and *Listeria monocytogenes*) are able to utilize both ethanolamine and 1,2-propanediol as a sole carbon source [37]. Recently, Kendall *et al.* (2012) suggested not only that ethanolamine utilization might provide a competitive advantage to enterohaemorrhagic *Escherichia coli* O157 but also that ethanolamine might act as a signal to initiate for expression of important virulence genes [38]. It is possible that we have identified some of the early signalling events and transcriptome changes associated with adaptation of *netB*-positive *C. perfringens* to the intestinal environment and/or specific environmental modifications. These results support the idea of the role of ethanolamine as a carbon and/or nitrogen source contributing to pathogenesis of intestinal infection as been recognized in promoting successful intestinal colonization by *C. difficile* [39].

It would be interesting to investigate further *C. perfringens* gene expression profiles during the different stages of NE in vivo. Further work is required to increase the relevance of findings of the ligated intestinal chicken loop model with clinical NE. Our analysis suggests that we may have identified early transcription events that initiate NE infection, since the chicken model as used did not show the transcription of main virulence genes. Infection for 8 h has been reported to induce classic lesions of NE [40], which might follow expression of a different group of genes including most virulence genes. A different approach may be examining the transcriptome in *C. perfringens* isolated directly of experimentally induced cases of NE, similarly described for *Campylobacter jejuni* [41]. Another possible approach is the examination of different in vitro conditions (temperature, carbon source, intestinal mucus, bile). Taking advantage of the transcriptome method at different stages of the pathogenesis of NE is very important to understanding the coordination of gene expression and the pathogenesis of the infection.

## Conclusions

This is the first transcriptome study using RNASeq on NE *C. perfringens* under in vivo and in vitro conditions, which demonstrate that the global gene expression were highly modulated by environmental conditions. Comparison of the global transcriptome response of in vivo to in vitro data revealed that the majority of virulence genes were least expressed in poor nutritional growth conditions and performed similarly to those expressed under 4-h in vivo conditions. We identified transcriptional regulators that play an important role in the adaptation of *C. perfringens*. Our data highlight the importance of understanding the gut microenvironment conditions that promote expression/repression of virulence genes in the NE pathogenesis.

## Abbreviations

BLAST, Basic Local Alignment Search Tool; COGs, Clusters of Orthologous Groups; DEGs, differentially expressed genes; FDR, false discovery rate; GO, Gene Ontology; H&E, hematoxylin and eosin; IV, in vivo; MDS, multi-dimensional scaling; mRNA, messenger RNA; NE, Necrotic enteritis; NELoc, Necrotic enteritis Locus; OS, osmotic shock; PCA, principal component analysis; PCR, polymerase chain reaction; PM, nutrient-poor media; qRT-PCR, Quantitative Real-Time PCR; RM, rich media; RNASeq, RNA sequencing method; RT, room temperature; TCS, two-component signal systems; TPG, tryptone-protease peptone-thioglycollic acid broth

## Additional files

Additional file 1: Table S1. Gene-specific primers for Quantitative Real-Time PCR (qRT-PCR)-sheet:AddTable01-primers and validation of RNAseq-sheet:AddTable01-qRT-PCRvsRNASeq. (XLSX 36 kb) Additional file 2: Figure S1. Volcano plots providing the fold change (log2) against the *p*-value (−log2) for all gene transcripts of NE *C. perfringens* CP1 in different growth conditions. Each dot corresponds to a specific gene. The dotted line represents the *p*-value ≤ 0.01, genes that are above, and farther away from the *p*-value line are highly significant when compared with the dots that are closer to the *p*-value line. Significantly differential transcript abundances of virulence-associated genes are highlighted in colors (red: NELoc1; green: NELoc2; blue: NELoc3; pink: QS; turquoise: 2CS; yellow: chromosomal toxins; orange: VR10B region; dark green: regulators and navy blue: plasmid). Genes plotted in the gray area are considered as no differentially expressed. (DOCX 1321 kb) Additional file 3: Table S2. Total number of NE *C. perfringens* CP1 strain genes significantly differentially expressed genes by RNASeq in different comparisons IV vs RM, IV vs PM, IV vs OS, PM vs RM, OS vs RM. (XLSX 200 kb) Additional file 4: Table S3. Genes significantly differentially expressed of the virulome of NE *C. perfringens* CP1 strain. (XLSX 38 kb) Additional file 5: Table S4. Genes significantly differentially expressed of the Two-Component system (TCS) of NE *C. perfringens* CP1 strain. (XLSX 42 kb) Additional file 6: Table S5. The total number of differentially expressed genes of IV vs RM (673), IV vs PM (382), IV vs OS (521) and PM vs RM (600) classified in three different GO terms: biological process (BP), molecular function (MF) and cellular component (CC). (XLSX 64 kb)
