# Supplementary material for: Comparative transcriptome analysis by RNAseq of necrotic enteritis Clostridium perfringens during in vivo colonization and in vitro conditions
Source: BMC Microbiol. 2016 Aug 12;16:186. doi: 10.1186/s12866-016-0792-6 (PMC4983038; doi:10.1186/s12866-016-0792-6)

Figure S1. Volcano plots providing the fold change (log2) against the p-value (-log2) for all gene transcripts of NE *C.perfringens* CP1 in different growth conditions. Each dot corresponds to a specific gene. The dotted line represents the p-value ≤ 0.01, genes that are above, and farther away from the p-value line are highly significant when compared with the dots that are closer to the p-value line. Significantly differential transcript abundances of virulence-associated genes are highlighted in colors (red:NELoc1; green;NELoc2; blue:NELoc3; pink: QS; turquoise:2CS; yellow: chromosomal toxins; orange:VR10B region; darkgreen: regulators and navy blue:plasmid). Genes plotted in the gray area are considered as no differentially expressed.


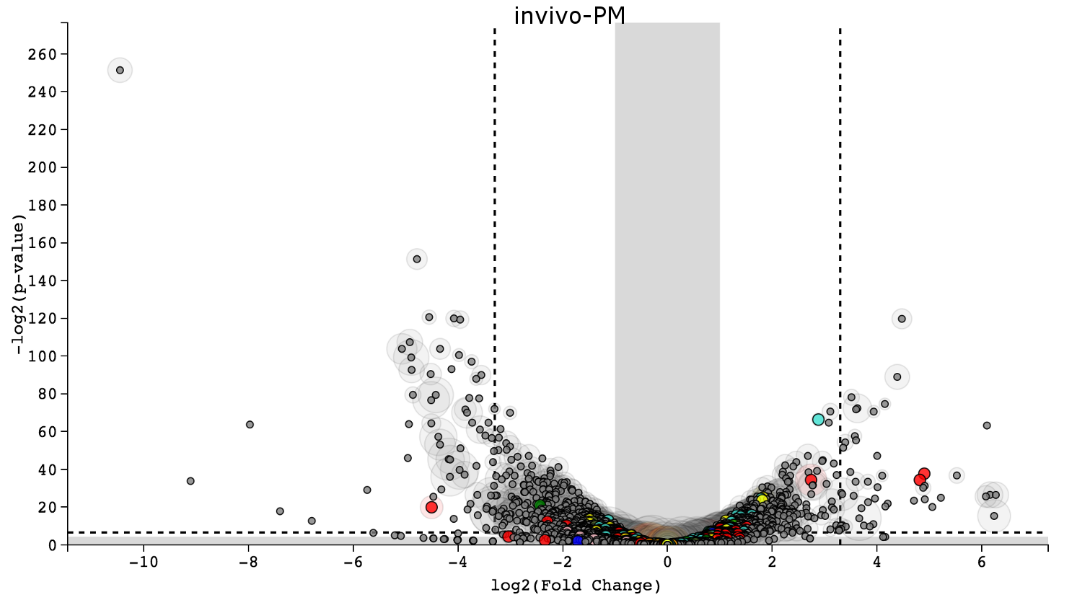


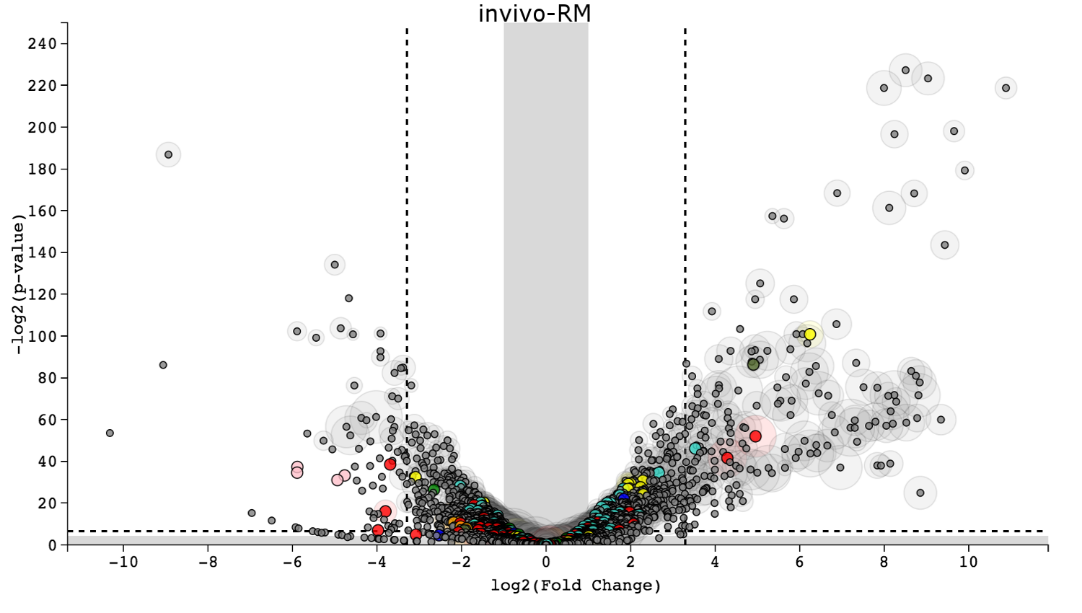


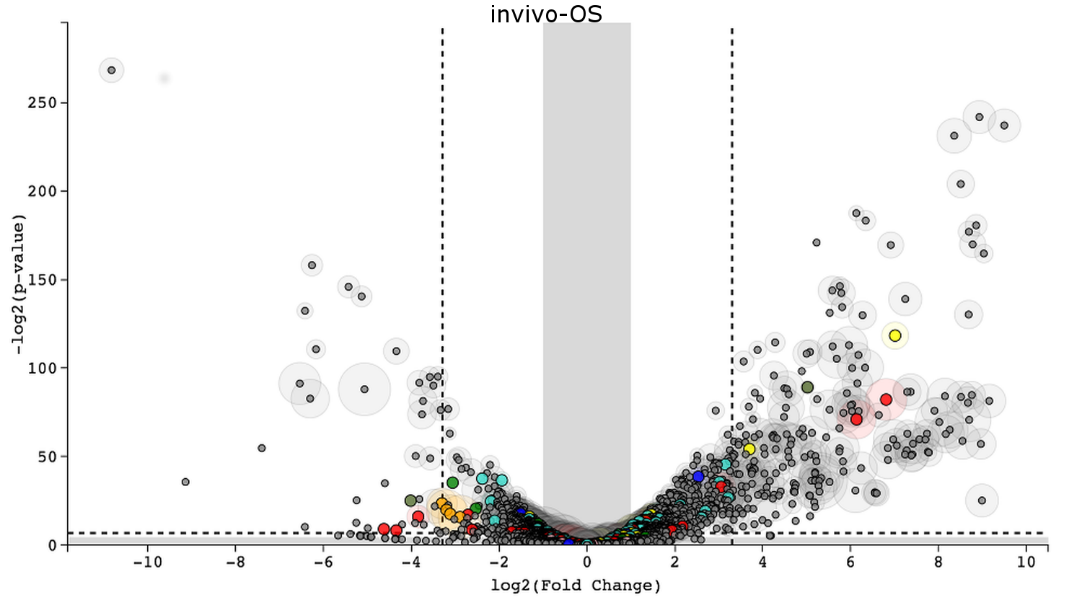


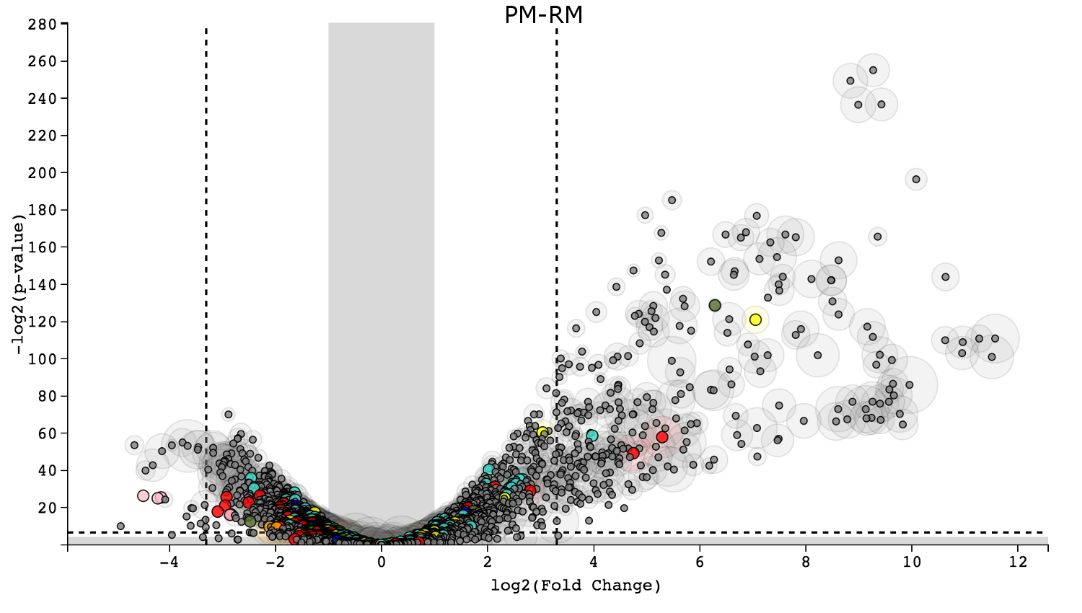


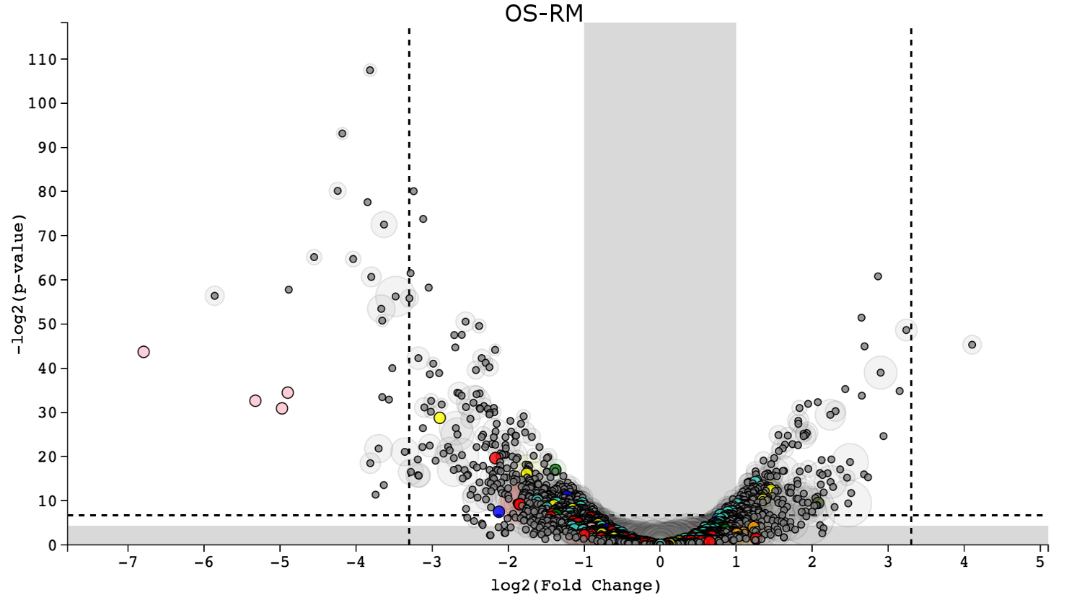

Supplement: Additional file 2: Figure S1. — Volcano plots providing the fold change (log2) against the p-value (−log2) for all gene transcripts of NE C. perfringens CP1 in different growth conditions. Each dot corresponds to a specific gene. The dotted line represents the p-value ≤ 0.01, genes that are above, and farther away from the p-value line are highly significant when compared with the dots that are closer to the p-value line. Significantly differential transcript abundances of virulence-associated genes are highlighted in colors (red: NELoc1; green: NELoc2; blue: NELoc3; pink: QS; turquoise: 2CS; yellow: chromosomal toxins; orange: VR10B region; dark green: regulators and navy blue: plasmid). Genes plotted in the gray area are considered as no differentially expressed. (DOCX 1321 kb) [file 12866_2016_792_MOESM2_ESM.docx]
